# Supplementary material for: Distrust within protected area and natural resource management: A systematic review protocol
Source: PLoS One. 2022 Mar 17;17(3):e0265353. doi: 10.1371/journal.pone.0265353 (PMC8929689; doi:10.1371/journal.pone.0265353)
Supplement: S1 File — (DOCX) [file pone.0265353.s002.docx]

# S1 File. Search string details for each database.

# Academic database search strategies

## Web of Science

Because of the restrictions on strings in the search, we will do each keyword group (distrust, NRM, protected areas) separately.

### Search 1

Core collection (Indexes**:** SCI-EXPANDED, SSCI, A&HCI, ESCI)

**All fields** = ("distrust*" OR "mistrust*" OR “untrust*” OR "cynic*" OR "skeptic*" OR “sceptic*” OR “lack of trust” OR “no* trust*” OR “absence of trust”)

### Search 2

Core collection (Indexes**:** SCI-EXPANDED, SSCI, A&HCI, ESCI)

**All fields** = (“natural resource management” OR “fisher* management” OR “wildlife management” OR “rangeland* management” OR “forest management” OR “land management” OR “catchment management” OR “freshwater management” OR “coastal management” OR “marine management” OR “ecosystem-based management” OR “ecosystem management” OR “environmental management” OR “collaborative management” OR “co-management” OR “adaptive management” OR “spatial management”)

### Search 3

Core collection (Indexes**:** SCI-EXPANDED, SSCI, A&HCI, ESCI)

**All fields** = (“protected area*” OR “protected forest*” OR “protected landscape*” OR “protected seascape*” OR “special protection area*” OR “special area* of conservation” OR “conservation area*” OR “wilderness area*” OR “management area*” OR “national heritage area*” OR “biodiversity area*” OR “bird area*” OR “locally managed marine area*” OR “indigenous and community conserved area*” OR “nature reserve*” OR “marine reserve*” OR “forest reserve*” OR “reserved forest*” OR “biosphere reserve*” OR “conservation reserve*” OR “community reserve*” OR “private reserve*” OR “national park*” OR “natural monument*” OR “national heritage place*” OR “wildlife sanctuar*” OR “national estuarine research reserve*” OR “communal forest*” OR “conservation zone*” OR “spatial closure” OR “spatial plan*”)

Then, we will combine searches with “AND” or “OR” as needed under the search history tab. Combine search 2 OR search 3, creating set #4. Then, combine #1 AND #4.

## Proquest Databases

Within ProQuest databases, I will search “anywhere except full text (NOFT)” instead of full text. There are multiple reasons for using the “anywhere except full text” restriction in ProQuest. The first is that it makes the search more similar to the search performed in Web of Science. Web of Science does not have full-text searching, so the number of results returned in Web of Science will always be lower in comparison to a database using full-text searching. Another reason for using the NOFT field in ProQuest is to reduce the noise in a systematic search where you want to (presumably) know if the purpose, methods, and findings of the sources you are examining are about your key topic areas. If so, your key topics should be represented in the title, abstract, keywords, and subjects of the records of those sources. Searching the full-text adds in noise because a source can refer to many issues in passing that are not the key focus of the source itself (mentions that might show up in the literature review, for example).

### Aquatic Sciences and Fisheries Abstracts

**Anywhere except full text (NOFT)** = (("distrust*" OR "mistrust*" OR “untrust*” OR "cynic*" OR "skeptic*" OR “sceptic*” OR “lack of trust” OR “no* trust*” OR “absence of trust”) AND ((“natural resource management” OR “fisher* management” OR “wildlife management” OR “rangeland* management” OR “forest management” OR “land management” OR “catchment management” OR “freshwater management” OR “coastal management” OR “marine management” OR “ecosystem-based management” OR “ecosystem management” OR “environmental management” OR “collaborative management” OR “co-management” OR “adaptive management” OR “spatial management”) OR (“protected area*” OR “protected forest*” OR “protected landscape*” OR “protected seascape*” OR “special protection area*” OR “special area* of conservation” OR “conservation area*” OR “wilderness area*” OR “management area*” OR “national heritage area*” OR “biodiversity area*” OR “bird area*” OR “locally managed marine area*” OR “indigenous and community conserved area*” OR “nature reserve*” OR “marine reserve*” OR “forest reserve*” OR “reserved forest*” OR “biosphere reserve*” OR “conservation reserve*” OR “community reserve*” OR “private reserve*” OR “national park*” OR “natural monument*” OR “national heritage place*” OR “wildlife sanctuar*” OR “national estuarine research reserve*” OR “communal forest*” OR “conservation zone*” OR “spatial closure” OR “spatial plan*”)))

### Agriculture and Environmental Sciences Collection

**Anywhere except full text (NOFT)** = (("distrust*" OR "mistrust*" OR “untrust*” OR "cynic*" OR "skeptic*" OR “sceptic*” OR “lack of trust” OR “no* trust*” OR “absence of trust”) AND ((“natural resource management” OR “fisher* management” OR “wildlife management” OR “rangeland* management” OR “forest management” OR “land management” OR “catchment management” OR “freshwater management” OR “coastal management” OR “marine management” OR “ecosystem-based management” OR “ecosystem management” OR “environmental management” OR “collaborative management” OR “co-management” OR “adaptive management” OR “spatial management”) OR (“protected area*” OR “protected forest*” OR “protected landscape*” OR “protected seascape*” OR “special protection area*” OR “special area* of conservation” OR “conservation area*” OR “wilderness area*” OR “management area*” OR “national heritage area*” OR “biodiversity area*” OR “bird area*” OR “locally managed marine area*” OR “indigenous and community conserved area*” OR “nature reserve*” OR “marine reserve*” OR “forest reserve*” OR “reserved forest*” OR “biosphere reserve*” OR “conservation reserve*” OR “community reserve*” OR “private reserve*” OR “national park*” OR “natural monument*” OR “national heritage place*” OR “wildlife sanctuar*” OR “national estuarine research reserve*” OR “communal forest*” OR “conservation zone*” OR “spatial closure” OR “spatial plan*”)))

## EBSCOhost Databases

We will search all EBSCOhost databases (Agricola; Fish, Fisheries, & Aquatic Biodiversity Worldwide; GreenFILE; and Wildlife & Ecology Studies Worldwide) at once and will a dd results to a folder (1-50, 51-100, etc.).

**All Text Fields (TX)** = (("distrust*" OR "mistrust*" OR “untrust*” OR "cynic*" OR "skeptic*" OR “sceptic*” OR “lack of trust” OR “no* trust*” OR “absence of trust”) AND ((“natural resource management” OR “fisher* management” OR “wildlife management” OR “rangeland* management” OR “forest management” OR “land management” OR “catchment management” OR “freshwater management” OR “coastal management” OR “marine management” OR “ecosystem-based management” OR “ecosystem management” OR “environmental management” OR “collaborative management” OR “co-management” OR “adaptive management” OR “spatial management”) OR (“protected area*” OR “protected forest*” OR “protected landscape*” OR “protected seascape*” OR “special protection area*” OR “special area* of conservation” OR “conservation area*” OR “wilderness area*” OR “management area*” OR “national heritage area*” OR “biodiversity area*” OR “bird area*” OR “locally managed marine area*” OR “indigenous and community conserved area*” OR “nature reserve*” OR “marine reserve*” OR “forest reserve*” OR “reserved forest*” OR “biosphere reserve*” OR “conservation reserve*” OR “community reserve*” OR “private reserve*” OR “national park*” OR “natural monument*” OR “national heritage place*” OR “wildlife sanctuar*” OR “national estuarine research reserve*” OR “communal forest*” OR “conservation zone*” OR “spatial closure” OR “spatial plan*”)))

Find all my search terms; uncheck “apply related words”; uncheck “apply equivalent subjects”; all publication types.

## PsycInfo (APA PsychNet)

**Any Field** = (("distrust*" OR "mistrust*" OR “untrust*” OR "cynic*" OR "skeptic*" OR “sceptic*” OR “lack of trust” OR “no* trust*” OR “absence of trust”) AND ((“natural resource management” OR “fisher* management” OR “wildlife management” OR “rangeland* management” OR “forest management” OR “land management” OR “catchment management” OR “freshwater management” OR “coastal management” OR “marine management” OR “ecosystem-based management” OR “ecosystem management” OR “environmental management” OR “collaborative management” OR “co-management” OR “adaptive management” OR “spatial management”) OR (“protected area*” OR “protected forest*” OR “protected landscape*” OR “protected seascape*” OR “special protection area*” OR “special area* of conservation” OR “conservation area*” OR “wilderness area*” OR “management area*” OR “national heritage area*” OR “biodiversity area*” OR “bird area*” OR “locally managed marine area*” OR “indigenous and community conserved area*” OR “nature reserve*” OR “marine reserve*” OR “forest reserve*” OR “reserved forest*” OR “biosphere reserve*” OR “conservation reserve*” OR “community reserve*” OR “private reserve*” OR “national park*” OR “natural monument*” OR “national heritage place*” OR “wildlife sanctuar*” OR “national estuarine research reserve*” OR “communal forest*” OR “conservation zone*” OR “spatial closure” OR “spatial plan*”)))

# Grey Literature Search Strategy

## CGIAR

<https://www.cgiar.org/research/publications/>

Enter distrust synonyms in the search box. Do not use “From” or “Research themes” filters. Search separately for each of the following words (using quotation marks): "distrust," "mistrust," “untrust,” "cynic," "skeptic," “sceptic,” “lack of trust,” “no trust,” “not trust,” and “absence of trust.”

## Center for International Forestry Research

<https://www.cifor.org/knowledge/publications/>

Enter distrust synonyms into the “search content” box. Filter by type “publication.” Do not use additional filters. Search separately for each of the following words (using quotation marks): distrust, mistrust, untrust, cynic, skeptic, sceptic, lack of trust, no trust, not trust, and absence of trust.

## Integrated Resource Management Applications: NPS

<https://irma.nps.gov/DataStore/Search/Advanced>

Search “any text field” for: "distrust" OR "mistrust" OR “untrust” OR "cynic" OR "skeptic" OR "sceptic" OR "lack of trust" OR "absence of trust" OR "no trust" OR “not trust.”

## NOAA Institutional Repository

<https://repository.library.noaa.gov/advancesearch>

Search Full text = ("distrust" OR "mistrust" OR “untrust” OR "cynic" OR "skeptic" OR "sceptic" OR "lack of trust" OR "absence of trust" OR "no trust" OR “not trust”).

## Treesearch: USDA Forest Service

<http://www.treesearch.fs.fed.us/>

Enter distrust synonyms into the “search content” box. Do not use additional filters. Search separately for each of the following words (using quotation marks): distrust, mistrust, untrust, cynic, skeptic, sceptic, “lack of trust,” “no trust,” “not trust,” and “absence of trust.”

## USFWS National Digital Library

<https://digitalmedia.fws.gov/digital/search/advanced/>

Enter distrust synonyms into the “search term” box. Search All fields for exact phrase. Do not use additional filters. Search separately for each of the following words (using quotation marks): distrust, mistrust, untrust, cynic, skeptic, sceptic, “lack of trust,” “no trust,” “not trust,” and “absence of trust.”

## World Bank

<https://openknowledge.worldbank.org/>

Enter distrust synonyms into the search box, searching “all of the OKR.” Search separately for each of the following words (using quotation marks): distrust, mistrust, untrust, cynic, skeptic, sceptic, “lack of trust,” “no trust,” “not trust,” and “absence of trust.” After, use relevant topic filters to narrow.

## WorldFish

<https://www.worldfishcenter.org/publications/search/advanced>

Enter distrust synonyms into the “search term” box. Search separately for each of the following words (using quotation marks): distrust, mistrust, untrust, cynic, skeptic, sceptic, “lack of trust,” “no trust,” “not trust,” and “absence of trust.”
